# Development of Food Multi-Mix Using a Linear Programming Approach to Fill the Nutrient Gap of Amino Acids and Micronutrients for Stunted Non-Wasted Children

**DOI:** 10.3390/foods12010064

**Published:** 2022-12-23

**Authors:** Nia N Wirawan, Umi Fahmida, Ratna C Purwestri, Ina S Timan, Badriul Hegar

**Affiliations:** 1 Doctorate Study Program in Nutrition, Department of Nutrition, Faculty of Medicine, Universitas Indonesia, Jakarta 10430, Indonesia; 2 Department of Nutrition, Faculty of Health Sciences, Universitas Brawijaya, Malang 65151, Indonesia; 3 Southeast Asian Ministers of Education Organization Regional Center for Food and Nutrition (SEAMEO RECFON), Pusat Kajian Gizi Regional Universitas Indonesia, Jakarta 10430, Indonesia; 4 Department of Excellent Research EVA 4.0, Faculty of Forestry and Wood Sciences, Czech University of Life Sciences Prague, Kamycka 129, 16500 Praha-Suchdol, Czech Republic; 5 Department of Clinical Pathology, Faculty of Medicine, Universitas Indonesia, Jakarta 10430, Indonesia; 6 Department of Pediatric and IMERI, Faculty of Medicine, Universitas Indonesia, Jakarta 10430, Indonesia

**Keywords:** food formulation, food multi-mix, linear programming, complementary feeding, amino acid, stunted, wasted, children

## Abstract

Food-based approaches using locally available food escalates the feasibility and the sustainability of nutrition intervention. A complementary feeding recommendation (CFR) integrated with the food multi-mix (FMM) formulation was targeted to fulfill micronutrient and amino acid requirements for stunted non-wasted (SNW) children aged 12–23 months living in agricultural areas. A seven-day estimated food record (EFR) of 87 children was used to design the CFR and 4 identified underutilized foods were integrated as the FMM. A linear programming approach using Optifood was applied to optimize the CFR and FMM. CFR alone successfully fulfills the vitamin C, riboflavin, iron, and zinc, but it cannot fulfill calcium, thiamin, niacin, vitamin B6, folate, vitamin B12, and histidine. With the incorporation of the selected underutilized cowpea, buncis batik, wader fish, and cows’ milk in the FMM development, the nutrients that are challenging in CFR development, can be fulfilled. Therefore, these findings present evidence that food multi-mix developed based on locally available nutrient-dense food sources can help to meet the nutrient gaps, which often remained even after a complementary feeding diet is optimized. Efficacy study using the developed CFR and FMM is recommended to assess effect in improving intake of micronutrients and amino acids and improving the linear growth of stunted, non-wasted children.

## 1. Introduction

Stunting is defined as low height for age z-score (HAZ) according to the World Health Organization [WHO] child growth standards 2005 [1]. Globally, approximately one-quarter of children are stunted [2,3] and almost all stunted children are living in developing countries [3]. In 2010, the prevalence of stunting in Indonesia was 35.6%, and it increased by 2% in 2013 [4] or become 36,4% in 2013 based on the Global Nutrition Report 2015 [5]. Even though there is prevalence reduction in 2017 [6], to achieve the target of 40% reduction in 2025 requires a huge effort and the collaboration of all sectors.

The condition of stunting is mostly concurrent with other low nutrition indicators, such as wasting. Data from the most current survey in Indonesia 2021 [7]) found that among 24.4% children with a low height for age z-score (stunting), more than two thirds were also wasting. It means that stunting as the form of chronic malnutrition mostly coexist together with other types of malnutrition. Assessment on the factors associated with stunting is needed as the form of chronic malnutrition may overlap with the cause of acute malnutrition (e.g., wasting) [8]. Therefore, the results of comparing stunting without excluding the acute malnutrition with non-stunted children, may exaggerate the difference between these two groups. 

The period from 6 to 24 months of age is one of the most critical periods for linear growth, but at the same time stunting reaches the highest prevalence, due to high nutrients requirements but limited quality and quantity of complementary foods [9]. Complementary feeding intervention affect length-for-age Z-scores by 0.0–0.64 [9], 0.08–0.62 [10,11] and the most efficacious intervention increase growth by ±0.7 Z-scores, which translates to a modest 30% reduction in stunting [12]. Other existing nutrition intervention are dietary supplements and food fortification, which are considered to be effective to fulfill the nutrients requirement, however the overall efficacy of these programs remains debatable because of costs and sustainability. In addition, there are advantages in utilizing commonly accessible, affordable, locally available food sources and processing methods that are familiar and culturally appropriate for communities. There is also evidence that natural food sources of nutrients are better absorbed than expensive synthetic supplements [13]. 

Protein is one type of nutrient that has long been well known as the cause of stunting in developing countries since the mid-1970s [14]. However, many studies showed that protein intake was sufficient. The findings among Indonesian children aged 3–12 years [15], 0–18 years old [16], 1–3 years old [17] and data from some other studies in six low income countries [18] revealed that protein intake is adequate. This finding lead to the conclusion that growth restriction is not due to protein deficiency [19]. Since then, micronutrient received main attention for the past four decades [14] to improve the health and survival of young children in developing countries. Micronutrient supplementation or fortification successfully reduces morbidity and mortality in young children in developing countries [20], however, the effect of micronutrient supplementation on the improvement of linear growth showed unsatisfactory results [21,22,23], suggesting that some fundamental dietary nutrients are lacking.

Issues on the need to re-examine protein recently emerge after the paper of Semba [14,24] regarding the low circulating amino acid among stunted children, including nine essential amino acids (EAA), three conditionally essential amino acids (CEAA) and three non-essential amino acids (NEAA) is published. Other studies found that eight plasma free amino acid (PFAA) essential (all EAA except isoleucine) [17] and valine were significantly lower among stunted children. This finding triggers the assessment of amino acid intake of stunted children. 

However, to the authors knowledge, no studies thoroughly examined the amino acid intake and the food formulation specifically designed to fulfil amino acids. Therefore, in this study, we aimed to develop a complementary food recommendation (CFR) and food multi-mix (FMM) formulation to fulfill the micronutrient as well as amino acid gap by optimizing the use of high-quality amino acid source from locally available, nutrient-dense foods. 

## 2. Materials and Methods

### 2.1. Study Design and Population under Study

This study was conducted in the agricultural areas, in Malang district, East Java, Indonesia. This paper is part of a comparative cross-sectional study comparing the stunted non-wasted and non-stunted non-wasted children. The development of complementary feeding recommendation and food multi-mix formulation was made for stunted non-wasted (SNW) 12–23 months children. A total of 87 SNW children was recruited for the study with the inclusion criteria: length-for-age Z-score (LAZ) < −2.0 SD, normal weight-for-length Z-score (WLZ) (≥−2 SD to ≤+1 SD), singleton, full-term gestation, normal birth weight, living with mother, and not suffered from diarrhea at least in the last one month (as reported by the mothers). The child was healthy and not suffering apparent symptoms that may affect his or her dietary intake, and the child’s primary caregiver was available and agreed to participate in the study.

### 2.2. Ethics Statement

A research protocol of the present study was approved by the Ethics Committee of the Faculty of Medicine, Universitas Indonesia, No. KET-1186/UN2.F1/ETIK/PPM.00.02/2019 and was registered in clinicaltrial.gov with identifiers NCT04157413 and unique Protocol ID: l9-10-1169. Written informed consent was obtained from each participant’s parent or responsible guardian. 

### 2.3. Anthropometry

Nutritional status of the children was assessed by using anthropometrics measurement, including length and weight. Body weight was measured by using the electronic platform model weighing scale (SECA 803; SECA, Hamburg, Germany) and was recorded to the nearest 0.1 kg. The recumbent length of children was measured to the nearest 0.1 cm by using a SECA 210 length board [1].

### 2.4. Dietary Intake

The 7-day estimated food record (EFR) data was used to develop the complementary feeding recommendation. The EFR was collected on all days of the week. Caregivers were asked to record all foods and beverages consumed by the child and estimate the amount in local cups or utensils in the provided form with the supervision of the field staff who visited the child’s house 5–6 times during the study. The visit aimed to validate the record by duplicating the food and or food weighing using an electronic kitchen scale (CAMRY, Guangdong, China, Model EK3131, precision ±2 g) and record the results. Field staff also recorded all composite foods and dishes and broke them down into their ingredients based on the recipe. Food models, food pictures and eating utensils were used in estimating the portion size. The food model used is produced by the National Institute for Health Research and Development Center Ministry of Health, in Bogor, Indonesia, and the food picture was developed from the earlier weighed food record study [25]. 

Before the dietary data collection, a group training session consisting of 5–7 mothers per session was applied to standardize the portion size. Every mother attended one time training and in total 20 training sessions covering the 12 villages were implemented. Every mother was requested to bring their child’s eating utensils, including a spoon, cup, bowl, plate, glass, bottle, etc. During the training, by using their child’s eating utensils, each mother was requested to demonstrate the average portion of child food consumption for each food item using the provided cooked foods. The food was then weighed and recorded on the flip sheet. At the end of the session, an agreement of the standardized spoon, cup, bowl, plate, glass, and bottle was made.

### 2.5. Food Composition Database

The food composition table was based on the Indonesian [26], the United States Department of Agriculture (USDA) and Vietnamese food composition tables. The amino acid composition was also obtained from the Japan food composition table. Nutrient composition for packaged foods was estimated from the nutrition facts and for the mixed food from the standard recipes. In some cases, the quantity of foods served and consumed was converted to their raw form using cooked to raw conversion factors to match nutrient values in food composition databases [27]. To avoid overestimation, nutrient values for the foods consumed in the cooked state were adjusted for cooking losses using USDA retention factors [28]. The specific food code allows for standardization for all data entry staff to choose the food. 

### 2.6. Statistical Analysis

Descriptive data of the respondents were presented as N (%), mean ± SD for normally distributed data or median (25th, 75th percentile) for not-normally distributed data. Data were analyzed using IBM SPSS statistic software version 20.0, Chicago, IL, USA.

### 2.7. Preparation of Model Parameters

Data from the dietary assessment survey defined the model parameters. The preparation of linear programming model parameters was done in Microsoft Excel 2013, Washington DC, USA. Serving size of each food item was defined as the 50th percentile, whereas the lower and upper limits of food pattern (servings per week) for the food items, food subgroups, and food groups were defined as the 5th, and 95th percentile. For the food group, median food pattern (50th percentile) was used as reference for the Best Diet Food Pattern (FP). The included food items in the models were foods that were consumed by ≥5% of children or consumed by <5% of the children but were considered nutrient-dense foods. 

### 2.8. Linear Programming Analysis

Development of CFR was performed by Optifood Software 2007, WHO/LSHTM/FANTA. Target nutrient for first and second run was presented in Table 1. The first run of the linear programming analysis (LPA) was done to optimize the fulfilment of default macro and micronutrients, including protein, fat, calcium, vit c, thiamin, riboflavin, niacin, vit b-6, folate, vit b-12, vit A, iron and zinc. The second run of the LPA was done to optimize the fulfilment of amino acids. In the second run of the LPA, default nutrients were replaced with the amino acids except for the energy, protein, fat and carbohydrate which were kept in the LPA. In the LPA, the nutrient goal for nutrients was based on the Indonesian recommended dietary allowance (RDA), whereas for amino acid it was based on the amended values from the 2007 WHO/FAO/UNU Report according to prescribed body weight [29]. The third run optimized amino acid using the additional 50% requirement. 

The result of LPA was presented as the %RNI from the no food pattern (NFP), i.e., the best diet (identified draft recommendation/optimal-case scenario) that can be deviate away or being optimized from the average food pattern but remains within upper and lower group constraint. If the %RNI from NFP is <100%, it means that the nutrient is the problem nutrient. The %RNI fulfilment that can be achieved when the diet is minimized (so called as the worst-case scenario) was presented as minimum (Min), whereas “Max or maximum” reflect the %RNI fulfilment when the diet is maximized (best-case scenario).

Based on the LPAs, nutrient gap for each nutrient was identified as the difference between 65% RNI to %RNI when nutrient content is minimized (the worst-case scenario). These nutrient gaps were used as a target for the food multi-mix formulation done by linear programming in the Nutrisurvey Software 2004. 

**Figure 1:**
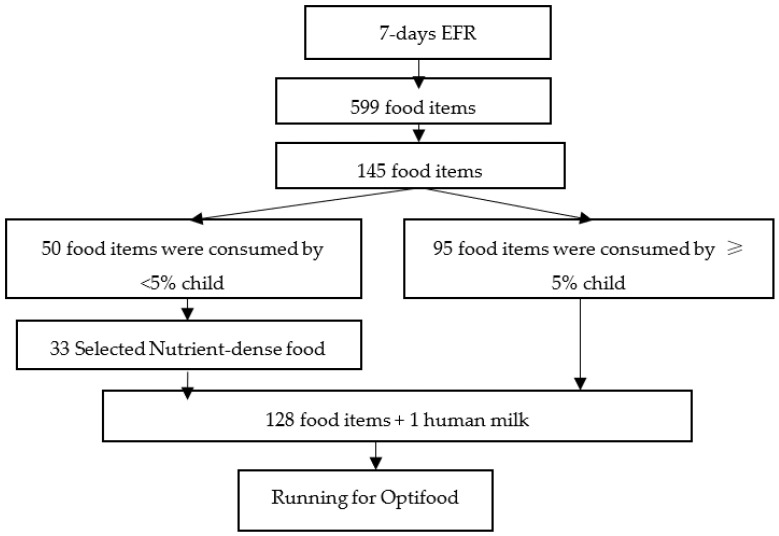
Number of Food Item Consume by Children and for Running the Optifood.

A total of 599 food items was identified from the 7 days EFR. After regrouping of similar food items, 145 food items remained. The 95 food items were consumed by ≥5% of the children and 33 food items were consumed by <5% of children but were considered as nutrient-dense food, therefore the 128 food items plus one human milk were included in the model. Number of food item consumed and selected for LPA was shown in Figure 1.

### 2.9. Food Multi-Mix Formulation 

Identified absolute problem nutrients or inadequate nutrients that cannot be fulfilled by the development of CFR, was covered by FMM. The steps of FMM development were:

1. Identification of locally available and culturally accepted food according to the results of dietary assessment and interview;
2. Processing of the identified food into powder or other intermediate form that include soaking, sprouting, heat treatment (boiling, steaming, pressure cooking) and fermentation depends on the identified food item;
3. Laboratory analysis;
4. Selection of the processed food into multi-mix based on the highest content of amino acid problem;
5. Formulation of most optimal composition of food item to be included in the FMM to fulfil nutrients gap by using the linear programming optimization in Nutrisurvey version 2004 software, Germany;
6. In setting the minimal goal of nutrient achievement, the biggest or highest gap, i.e., difference between 65% RNI to %RNI for nutrient when nutrient content was minimized (worst-case scenario) in the LPA was used (the lowest and the highest value shows the gap of RNI fulfillment. The lowest value was calculated from the gap between the 100%RNI with the % RNI achieved by the using the best diet NFP (best diet that are optimized from the average food pattern), whereas the highest nutrient gap shows the gap between the 65% RNI (the RNI should be achieved at least 65% from the diet) and the fulfillment of %RNI when nutrient content is minimized (worst-case scenario);
7. In addition, the ratio between animal to plant protein sources was targeted to meet the minimum of 4, based on a difference between stunted, non wasted vs. non stunted, not wasted children in the study.)

The FMM optimization in the Nutrisurvey Software 2004 was made with the following steps:

1. Inputting the selected potential food items i.e., soaked cowpeas and buncis batik, boiled wader fish and curd;
2. Inputting the target nutrient achievement based on nutrient gap;
3. Inputting the minimum and maximum amount (in grams) for each food item;
4. Calculating the most optimum formulation to achieve the target nutrients.

## 3. Results

### 3.1. General Characteristics and Nutritional Status of the Children

Total analyzed subjects were 87 from 2 subdistricts and 13 villages. The education level of father and mother was mainly graduated from elementary and junior high schools (>60%), even though some of the parents graduated from academy or university (4%). Type of household was equal between nuclear and extended families, with the average number of people living in a household being four. The main occupation of the father was mainly farmer (30%) and farm/livestock worker (23%). The classification of father’s income generating activity, 60% of fathers were in agricultural related activity (as owned/shared land farmer as worker). The average age of father was 31 years old, whereas mother was 26 years old. Total income per month from both father and mother, in average was 3.6 million IDR (USD 241.4). The median income was higher than the minimum district wages (2.781.564 IDR or 191.83 USD). Approximately 40% of the subjects owned their house. The mean age of the children was 16 months, with weight 8–9 kg, length 72 cm. The mean weight for length for age z-score (LAZ) was −2.5 SD and the weight for length z-score was −0.65 SD as shown in Table 2. 

### 3.2. Complementary Feeding Recommendation

The LPA showed that there was no absolute or partial problem nutrient but the energy, calcium, vitamin C, all B-vitamins (B1, B2, B3, B6, folate and B12), iron and zinc were inadequate. Using the current amino acids requirement, there was no problem nutrient or dietary inadequacy in stunted non-wasted children. However, after the use of 50% additional requirement for amino acids, histidine was identified as an absolute problem nutrient (Table 3).

The complementary feeding recommendation to fulfil micronutrients and amino acids were: (1) The consumption of grain and its product at least 3x/day; (2) Consume meat-fish-egg at least 3x/day including egg 1x/day and small fish with bones 4x/week; (3). Consume legumes-seed-nut including soybean and its product at least 2x/day; (4). Consume fruits at least 1xday including vitamin C and vitamin A rich fruit; (5). Consume vegetables 2x/day including dark green leafy vegetables; (6). Consume dairy product including fresh milk at least 4x/week; and (7). Continue breastfed children on demand. With this recommendation, it can fulfil Vit C, Riboflavin, Iron and Zinc. However, it cannot fulfil Ca, Thiamin, Niacin, B6, folate and B12 and histidine. The nutrients that cannot fulfil by CFR be the target nutrient of FFM as shown in Table 4.

### 3.3. Identification of Underutilized Locally Available Food

The underutilized locally available food was selected based on the following criteria: foods were grown/bred or could be found and consumed by the household in the study areas. These two criteria reflect that the food is available in the study area but were only consumed by few (<5%) respondents. Based on those considerations, three underutilized foods were identified, i.e., cowpea, *buncis tunggak* and *wader* fish (small freshwater fish with bone). *Wader* fish was selected due to the consideration that the study areas were located surrounded by Selorejo Dam, a tourism spot where wader fish was consumed by the visitors but were not sufficiently consumed by the local people particularly children. 

The food processing was performed before further processing into the powder form. Food Processing into intermediate product was described elsewhere. The assessment of nutrient content of the intermediate products was described elsewhere.

### 3.4. Food Multi-Mix

The selected food was soaked cowpea flour (SCF), soaked buncis batik flour (SBBF), boiled wader fish flour (BWFF) and Curd (CD), which had the highest histidine content. Five formulations of food multi mix (FMM1 to FMM5) were identified from LPA in Nutrisurvey. Out of these, FMM2, FMM3, FMM4, and FMM5 met the animal to plant protein ratio of 4 (Table 5).

## 4. Discussion

Using the linear programming approach, we identified that stunted, non-wasted children had dietary inadequacy in micronutrients (Ca, Fe, Zn, folate, B, C vitamins) but not amino acids. When amino acid requirements were increased by 50% for allowance of chronic undernutrition, histidine was an absolute problem nutrient. Based on the nutrient gaps of these micronutrients and amino acid histidine, several formulations of food multi mix were developed using locally available nutrient-dense foods. Inclusion of these FMMs with the CFR can help to meet the dietary adequacy of all nutrients, including the limiting amino acid histidine.

Previous studies using an LP approach to develop CFRs have shown that optimized CFRs can increase intake of problem nutrients. The developed CFRs for children in Lombok significantly increased intakes of calcium, iron, niacin, and zinc. The incorporation of shredded chicken liver/fish or dried anchovy in each main meal was designed to increase intakes of iron, zinc, and calcium. [32]. However, the CFR development in Ghana found that among the breastfed children, iron, calcium, and folate, zinc, vitamin A, riboflavin and vitamin C remained as dietary inadequate. For the non-breastfed 12–23-month-old children, nutrient requirements that could not be met in the optimized CFRs were iron, vitamin A, niacin, iron, folate, and calcium. A study in Tanzania [33] found that dietary adequacy for calcium was difficult to meet and in some settings were folate and B vitamins [18]. As a result, additional interventions are required to increase consumption of nutrient-dense foods beyond those that are already consumed by infants and young children from the complementary feeding [34].

The combination of CFR and FMM successfully fulfills the gap of nutrient inadequacy not only for macro and micronutrients but also for amino acids, including histidine, which was the absolute problem nutrients. The development of FMM aimed to achieve the biggest gap between 65% RNI and the worst-case scenario. Moreover, the ratio between animal to plant protein was also the key consideration in selecting the optimal formulation for FMM.

The identified locally available underutilized food included plant-protein and animal protein food source. Apart from the protein content in legumes, legumes were also found to have an important role in proper gut barrier and immune system development. In nutrition intervention, besides the adequacy of nutrients, the role of other bioactive compounds in plant food sources was emerging. Nutrient absorption is affected by dietary fiber through the presence of beneficial colonic microflora, which can lead to improved gut barrier function and decreased inflammation to reduce the risk of developing EED. Cowpea was found to have dietary fiber that potential to be incorporated in complementary food to promote gut health in children and in turn, will have better nutritional status [35,36].

High-quality proteins with balanced amino acid profiles in legumes are important for proper gut barrier and immune system development. It is known that EED both derives from and contributes to a disturbed microbiome and a dysfunctional gut immune system. The incorporation of common bean to complementary feeding of rural Malawian children during the second year of life led to an improvement in a biomarker of gut health, even though the direct effect on improvement of linear growth was not observed [37].

However, legumes also naturally contained anti-nutritional factors, such as phytate. Phytate is the storage form of phosphorus in plants and is found in high concentrations in seeds, cereals and pulses [38]. Traditional household processing methods such as fermentation, soaking [39], germination, and hydrothermal processing may reduce the phytate content of unrefined cereals and legumes through dephosphorylation of InsP6 to lower myo-inositol phosphate forms, some of which no longer inhibit the absorption of zinc (InsP4– InsP1) [40]. The pretreatment commonly used to reduce phytate in legumes food are soaking, sprouting and boiling. In our study we chose soaking as it is practical while giving comparable nutrient contents as compared to sprouting and boiling.

In the development of food multi-mix, inclusion of fish products, milk and milk products and/or other animal source foods can increase its protein quality [39]. The incorporation of flesh foods, such as dried whole fish with bones enhances micronutrient content and bioavailability in the daily diet [41]. In many countries, fish is a more feasible alternative than meat or poultry, especially in countries where economic, religious and/or cultural factors prevent the consumption of meat and poultry. Fish contains moderate levels of readily available heme iron; approximately 60 % of the iron in fish is highly bioavailable heme iron. Fish is also a relatively good source of fat, riboflavin, niacin, vitamin B12, calcium and provided it is consumed whole with bones also calcium and zinc [42,43]. Calcium absorption from small soft-boned fish eaten whole with the bones appears to be comparable to that of milk [44,45].

The formulated FMM4 and FMM5 can achieve the targeted ratio of animal to plant protein source, histidine, vitamin B2, B12 and folate. However, it did not fully meet adequacy of thiamin, niacin, B6, and calcium. Even though the wader fish have been incorporated in the FMM with the amount of 25–30 g, calcium adequacy was still not achieved.

Other findings revealed from this study support the previous suggestion that current estimates of protein and essential amino acid requirements need to be re-evaluated considering the repeated exposure to poor hygiene and sanitation and energy deficit in children in developing countries [29,30]. From studies of protein absorption in south Asia, where EED is common, it is estimated that the AA requirement is increased by 15% [46] or three-fold during inflammation [47]. A study among North American children found that the increased AA requirement caused by EED is multiplicative with the changes in AA requirements caused by rapid growth or acute infection [46]. A study among adults in India even found that higher levels of essential amino acids needed such as lysine up to 50% higher in chronically undernourished compared to well-nourished controls [30]. However, the effect of inflammation on protein and amino acid requirements among children in developing countries is poorly understood [48]. The current estimates of protein and essential amino acid requirements do not address the question of increased requirements due to frequent infections and energy deficits in children in developing countries [29,30].

To ensure the internal reliability of the study, the dietary intake for CFR development was derived from the non-consecutive 7-days EFR. At the final samples, all days of week was represented. The EFR was also validated with the 5–6 times visit of the field staff to validate the self-estimated food record made by the child’s caregiver by duplicating the food and or food weighing. Moreover, a group for training of mothers prior to data collection was made to standardize the portion size estimation using the child’s own eating utensils. The FCT was also carefully developed by ensuring the completeness of the nutrient content. When borrowing the nutrient content from other food composition tables, the water content and nutrient values for the foods consumed in the cooked state were adjusted for cooking losses using USDA retention factors [28]. The specific food code was also developed to allow standardization during data entry. Moreover, for packaged food, collection of nutrition facts was also done. For the mixed food, development of standard recipe to calculate the nutrient content was also applied. In some cases, the quantity of foods served and consumed was converted to their raw form using cooked to raw conversion factors to match nutrient values in food composition databases [27]. The selection of only non-wasted stunted children can be both the limitation and strength of this study. While it limits the interpretation to only a subgroup of stunted children, the selection of only SNW children allows for a clear understanding of what is occuring among stunted children by excluding the possibility of shared causal pathways with other forms of malnutrition. In addition, the laboratory analysis of identified underutilized locally available foods only assessed the content of amino acids and did not incorporate other nutrients, which can also be a limitation. The content of other nutrients was derived from the available databases. To the author’s knowledge, this is the first paper that utilized an LP approach to identify the problem nutrients of amino acids and to ensure the adequacy of the amino acids. Moreover, we combined the CFR with food-multi mix and describe how the combination of these two approaches successfully fulfills the nutrient requirement. In addition, the optimization of underutilized locally available food also supports the findings that in an agricultural area, there is potential of locally available nutrient-dense foods to solve malnutrition. We acknowledge the need to improve amino acid composition of foods in the Indonesian food composition table. The ratio of animal to plant protein food source needs to be considered in the development of a food-based approach for tackling stunting in young children. Finally, we recommend an efficacy study to test the developed CFR and FMM.

## 5. Conclusions

The combination of food multi-mix formulation by incorporating the locally underutilized available foods into the complementary feeding recommendation successfully fulfills the gap of nutrient inadequacy not only for macro and micronutrients but also for amino acids, including histidine, which was the absolute problem nutrients.

## Figures and Tables

**Table 1:** Nutrients requirement as the target of LPA.

Nutrients	Requirements	
Energy ^1^ (kcal)	1350	
Protein ^1^(gram)	20	
Fat ^1^ (gram)	30	
Calcium ^1^ (mg)	650	
Vitamin C ^1^ (mg)	40	
Thiamin ^1^ (mg)	0.5	
Riboflavin ^1^ (mg)	0.5	
Niacin ^1^ (mg)	6	
Vitamin B-6 ^1^ (mg)	0.6	
Folate ^1^ (mcg)	160	
Vitamin B-12 ^1^ (mcg)	1.5	
Vit A (RE) ^1^	400	
Iron ^1^ (mg)	7	
Zinc ^1^ (mg)	3	
Isoleucine (mg/d)	351 ^a^	526.5 ^b^
Leucine (mg/d)	702 ^a^	1053 ^b^
Lysine (mg/d)	572 ^a^	858 ^b^
SAA ^2^ (methionine and cysteine) (mg/d)	286 ^a^	429 ^b^
AAA ^3^ (phenylalanine + tyrosine) (mg/d)	520 ^a^	780 ^b^
Threonine (mg/d)	312 ^a^	468 ^b^
Tryptophane (mg/d)	83.2 ^a^	124.8 ^b^
Valine (mg/d)	468 ^a^	702 ^b^
Histidine (mg/d)	195 ^a^	292.5 ^b^

^1^ Based on Indonesian recommended daily allowance 2019 for children 1–3 years; ^2^ SAA= Sulphur Amino Acid (meth + Cys); ^3^ AAA= Aromatic Amino Acid (pen + tyr); ^a^ Requirement based on Amended Values from the 2007 WHO/FAO/UNU Report) using prescribed body weight of 13 kg (according to Indonesian recommended daily allowance 2019 for children 1–3 years); ^b^ Higher levels of essential amino acids up to 50% [30].

**Table 2:** General Characteristic of the Subjects.

Characteristics	N = 87
Father’s education level ^1^	
illiterate	2 (2.3)
Elementary school	33 (37.9)
Junior high school	32 (36.8)
Senior high school	17 (19.5)
Academy/University	3 (3.4)
Mother’s education level ^1^	
Illiterate	1 (1.1)
Elementary school	24 (27.6)
Junior high school	44 (50.6)
Senior high school	16 (18.4)
Academy/University	2 (2.3)
Types of HH ^1^: Nucleus family	46 (52.9)
Child’s sex ^1^: Boys	45 (51.7)
Father’s main occupation ^1^	
Farmer (Owner/shared land)	27 (31)
Breeder (cow/chicken/duck)	6 (6.9)
Fisherman	1 (1.1)
Farm/livestock worker	24 (27.6)
Daily worker non-farm/livestock (construction, industry, driver)	10 (11.5)
Employee	7 (8)
Entrepreneur	11 (12.6)
Main income from agricultural activities	58 (66.7)
Father’s age (year) ^2^	31.7 ± 6.6
Mother’s age (year) ^2^	26.1 ± 5.6
Number of people living in a HH ^2^	4 (4; 6)
Number of people in HH who earn money ^2^	2 (1; 3)
Total income from father and mother ^2^ (thousand rupiahs)	2320 (1400; 3760)
House ownership ^1^	32 (36.8)
HH financial management by mothers ^1^	77 (88.5)
Child’s age	16.5 (13.7; 19.4)
Weight (kg)	8.45 ± 0.80
Length (cm)	72.96 ± 3.08
WLZ ^2^	−0.65 ± 0.73
LAZ ^2^	−2.54 (−2.82; −2.20)
WAZ ^2^	−1.74 ± 0.60

^1^ Presented as N (%), ^2^ Presented as Mean ± SD or Median (25th, 75th percentile).

**Table 3:** LP Output of stunted non-wasted children.

	First Run				Second Run ^5^				Third Run ^6^			
Nutrient	NFP ^1^	Min	Max	Note	NFP ^1^	Min	Max	Note	NFP ^1^	Min	Max	Note
Energy	100	31.7	136.5	Inadequate	100	31.6	115.6	Adequate	100.00	31.6	115.6	Inadequate
Protein	106.2	92.6	233	Adequate	208.2	139.9	234.3	Adequate	213.70	139.9	234.3	Adequate
Fat	100	78.6	152.1	Adequate	121.8	99.8	162.1	Adequate	135.00	99.8	162.1	Adequate
Ca	100	33.7	146.8	Inadequate								
Vit C	149.6	61	272.7	Inadequate								
Vit B1	102.2	33.3	223.7	Inadequate								
Vit B2	153.9	63.6	321.9	Inadequate								
Vit B3	115.5	38.6	228.7	Inadequate								
Vit B6	100	19.6	141.9	Inadequate								
Folate	102.2	23.1	222.6	Inadequate								
Vit B-12	104.5	23.9	161.7	Inadequate								
Vit A RE	491.8	258.1	980	Adequate								
Iron	100	44.1	191.5	Inadequate								
Zinc	134	58.1	258.8	Inadequate								
His					435.2	249.4	525.3	Adequate	34.6	16.6	35	Absolute PN ^4^
Lys					398.8	229	481.1	Adequate	313.8	152.7	320.8	Adequate
SAA ^2^					496.3	268.2	577.7	Adequate	366.8	178.8	385.1	Adequate
AAA ^3^					540.7	330.7	650.5	Adequate	427.6	220.4	433.7	Adequate
Thre					482.3	292.8	574.1	Adequate	376.0	195.2	382.8	Adequate
Tryp					544.3	350.1	657.7	Adequate	415.2	233.4	438.5	Adequate
Val					409.6	256.6	488.8	Adequate	319.3	171.1	325.9	Adequate
Leu					417.6	268.2	497.7	Adequate	325.5	178.8	331.8	Adequate
Isoleu					487.2	307.4	593.3	Adequate	379.9	204.9	395.5	Adequate

^1^ NFP = No food pattern; ^2^ SAA= Sulphur Amino Acid (meth + Cys); ^3^ AAA= Aromatic Amino Acid (pen + tyr); ^4^ PN = problem nutrient; ^5^ The amino acid goal was based on the current Amended Values from the 2007 WHO/FAO/UNU Report (with prescribed body weight of 13 kg (according to Indonesian recommended daily allowance 2019 for children 1–3 years); ^6^ The amino acid goal was based on the 50% additional requirement.

**Table 4:** Minimal Nutrient Target for FMM.

Nutrient	WHO/UNICEF 98	Nutrient Gap (% RNI)	
		Lowest ^1^	Highest ^2^
Energy (kcal)	746	33.3	246.18
Protein ^3^ (gram)	10.9	-	6
Thiamin (mg)	0.5	31.7	0.16
Riboflavin (mg)	0.6	1.4	0.19
Niacin (mg)	8	26.4	2.11
B6 (mg)	0.7	45.4	0.32
Folate (mcg)	50	41.9	20.95
B12 (mcg)	0.5	41.1	0.21
Vit C (mg)	30	4	1.20
Ca (mg)	350	31.3	109.55
Fe (mg)	6	20.9	1.25
Zn (mg)	2.8	6.9	0.19

^1^ lowest gap = %100 RNI–%RNI in best diet no-FP; ^2^ highest gap = 65% RNI–%RNI in the worst-case scenario; ^3^ protein is adequate (>65% RNI in worst-case scenario). However, in the FMM optimization, the minimum target was set based on guidelines for Indonesia National Standard (SNI) for complementary food [31].

**Table 5:** The Fulfillment of Nutrient Requirements by CFR and FMM.

Nutrient	Req ^1^	CFR ^2^		FMM ^3^					
		Med	Max	FMM0 ^4^	FMM1 ^5^	FMM2 ^6^	FMM3 ^7^	FMM4 ^8^	FMM5 ^9^
Energy (kcal)	746	412.5	834.5	241.1	246.3	245.4	246.6	277.7	245.9
Protein (g)	10.9	22.8	47.3	14.8	24.2	26.6	26.8	29.3	31.2
Vit. B1 (mg)	0.5	0.2	0.4	0	0.1	0.1	0.1	0.1	0
Vit B2 (mg)	0.6	0.5	0.9	0	0.1	0.1	0.1	0.2	0.4
Vit. B6 (mg)	0.7	0.4	0.9	0	0.1	0.1	0.1	0.1	0.1
Tot. fol.acid (mcg)	50	79.3	154.4	19.4	7.9	7.7	12.1	20.9	31
Vit. B12 (mcg)	0.5	1.5	2.6	0	0	0	0.1	0.3	0.7
Vit. C (mg)	30	38.2	63.8	0	0	0	0	0	0
Calcium (mg)	350	313.3	638.9	0	8	6.4	11.4	21.4	30
Iron (mg)	6	6.2	12.1	4.1	3.9	4.2	3.9	4.1	4.1
Zinc (mg)	2.8	1.6	3	2.5	7.8	8.7	8.7	8.9	8.8
Isoleucine (g)	0.53	1.1	2.3	0.7	1.1	1.2	1.3	1.4	1.5
Leucine (g)	1.05	1.9	3.9	1.2	2	2.2	2.3	2.5	2.7
Lysine (g)	0.86	1.5	3.2	0.7	1.5	1.6	1.7	1.9	2.1
SAA (g)	0.43	0.8	1.7	0	0.8	0.8	0.8	1	1.1
AAA (g)	0.78	1.8	3.7	2	3	3.3	3.3	3.5	3.7
Threonine (g)	0.47	1	2.1	0.9	1.3	1.5	1.5	1.6	1.7
Tryptophane (g)	0.12	0.3	0.5	0.2	0.2	0.2	0.2	0.2	0.3
Valine (g)	0.70	1.2	2.5	0.8	1.3	1.4	1.5	1.6	1.7
Histidine (g)	0.29	0.6	1.2	0.6	0.8	0.9	0.9	1	1
Arginine (g)	NA	1.4	3	1.1	1.9	2.1	2.1	2.3	2.3

^1^ Based on the requirements from complementary food for breastfed children (WHO/UNICEF 1998). Amino acid requirement was based on the WHO/FAO/UNU 2007 that has been added with 50%requirement for chronically undernourished children [30]; ^2^ FMM = food multi-mix; ^3^ CFR = Complementary feeding recommendation; ^4^ FMM0 (SCF 72 g); ^5^ FMM1 (SCF 5 g, SBBF 10 g, BWFF 25 g, CD 30 g, BRF 25 g); ^6^ FMM2 (SCF 5 g, SBBF 10 g, BWFF 30 g, CD 30 g, BRF 20 g); ^7^ FMM3 (SCF 1 g, SBBF 10 g, BWFF 30 g, CD 30 g, Egg 10 g, BRF 20 g); ^8^ FMM4 (SCF 1 g, SBBF 10 g, BWFF 30 g, CD 30 g, Egg 30 g, BRF 20 g); ^9^ FMM5 (SCF 1 g, SBBF 8 g, BWFF 30 g, CD 30 g, Egg 60 g); Soaked cowpea flour (SCF), Soaked buncis batik flour (SBBF), Boiled wader fish flour (BWFF), Curd (CD), Brown rice flour (BRF).

## Data Availability

The data presented in this study are available on request from the corresponding author.
